# Elemental Composition Analysis of Sheep Tears Using Particle‐Induced X‐Ray Emission

**DOI:** 10.1111/vop.70096

**Published:** 2025-10-01

**Authors:** Alon Zahavi, Oren Pe'er, Noa Cohen Sinai, Yoav Vardizer, Lionel Sebbag, Ron Ofri, Olga Girshevitz, Nitza Goldenberg‐Cohen

**Affiliations:** ^1^ Department of Ophthalmology and Laboratory of Eye Research, Felsenstein Medical Research Center Rabin Medical Center Petach Tikva Israel; ^2^ Faculty of Medicine Tel Aviv University Tel Aviv Israel; ^3^ Koret School of Veterinary Medicine Hebrew University of Jerusalem Rehovot Israel; ^4^ Ophthalmology Department Bnai Zion Medical Center Haifa Israel; ^5^ Institute of Nanotechnology and Advanced Materials Bar Ilan University Ramat Gan Israel; ^6^ Krieger Eye Research Laboratory, Rappaport Faculty of Medicine Technion‐Israel Institute of Technology Haifa Israel

**Keywords:** biomarker, elemental composition, PIXE, sheep, tears

## Abstract

**Objective:**

To identify the elemental composition of ovine tears using particle‐induced X‐ray emission (PIXE).

**Animals Studied:**

Ten physically healthy female sheep.

**Procedures:**

Tear samples were collected using Schirmer tear test strips. Tear samples were collected once daily over five consecutive days to assess reproducibility. Elemental analysis was performed with PIXE. Data were processed with GUPIX software. Median values of the elements identified were calculated and statistically analyzed with Kruskal–Wallis and post hoc Dunn's tests.

**Results:**

The analysis demonstrated consistent elemental concentrations over the 5‐day sampling period. Among the elements detected, chloride (Cl) and sodium (Na) exhibited the highest concentrations.

**Conclusions:**

This study establishes PIXE as a valuable technique for detecting and analyzing the elemental composition of the tear film in sheep. By providing a precise method for elemental analysis in small biological samples, this approach lays the groundwork for future research exploring its reliability and sensitivity in tear analysis and potential as a biomarker for ocular and systemic health. The impact of variables including age, diet, and environmental exposure on tear film constituents should be studied, potentially extending the methodology to other species.

## 1 Introduction

The tear film is vital for the maintenance of ocular surface homeostasis, providing lubrication, metabolic support, and protection of the cornea and conjunctiva. Its composition is affected by systemic disease, nutrition, and environmental factors and can reflect systemic concentrations of elements [1, 2, 3]. Although tear sampling is generally noninvasive and relatively simple, analysis of the components of the tear film is limited by its small volume [4, 5, 6, 7, 8, 9, 10, 11]. Previous studies demonstrated that the composition of the tear film in animals is unique for each species and correlated with their habitats [12, 13]. However, the analyses focused mainly on the protein, cholesterol, and glucose contents of the tears and did not address elemental composition. The elemental composition of the tear film may serve as a biomarker for monitoring ocular pathologies, systemic disease, and environmental pollution.

Recent technological advances have made it possible to apply ion accelerators that generate a MeV (Mega‐electron Volt) proton (H^+^) beam to biological samples. Particle‐induced X‐ray emission (PIXE) has proven to be an accurate and sensitive method for elemental analysis of small samples [14, 15]. High‐energy ions bombard the specimens, and the elements present are identified by the corresponding X‐ray energies emitted. Their concentrations are deduced from the X‐ray intensities. To date, only a few studies have used PIXE to analyze the elemental composition of ocular tissues [14, 15].

The purpose of this study was to identify the elemental composition of ovine tears using PIXE. Characterizing the elemental composition will improve our understanding of ocular pathophysiology and surface homeostasis in sheep and establish a methodology for tear film elemental analysis in other species.

## 2 Materials and Methods

### 2.1 Study Design and Population

The study was approved by the Institutional Animal Care and Use Committee and conducted in accordance with the Association for Research in Vision and Ophthalmology (ARVO) guidelines for the use of animals in research.

The study was performed at a university‐affiliated veterinary center in domesticated sheep. All sheep included in this study were female Assaf‐Afec crossbred sheep, born and raised at an experimental flock, where they were kept in a semi‐open shed facility with free access to outdoor air. The animals were housed on concrete flooring, not on pasture or dirt. All sheep were housed in the same environment and raised under the same habitat and dietary conditions. Prior to data collection, the animals underwent a complete physical examination by a specialist in small‐ruminant medicine and a comprehensive ophthalmic examination by a board‐certified veterinary ophthalmologist using slit‐lamp biomicroscopy (Kowa SL‐17 Portable Slit‐Lamp, Kowa Ophthalmic and Medical Equipment, Torrance, CA), rebound tonometry (TonoVet Plus, ICare Finland Oy, Vantaa, Finland), and indirect ophthalmoscopy (Welch Allyn, Skaneateles, NY). Animals were excluded if they had any ocular or systemic abnormalities or were uncooperative.

### 2.2 Tear Sampling Technique

**FIGURE 1:**
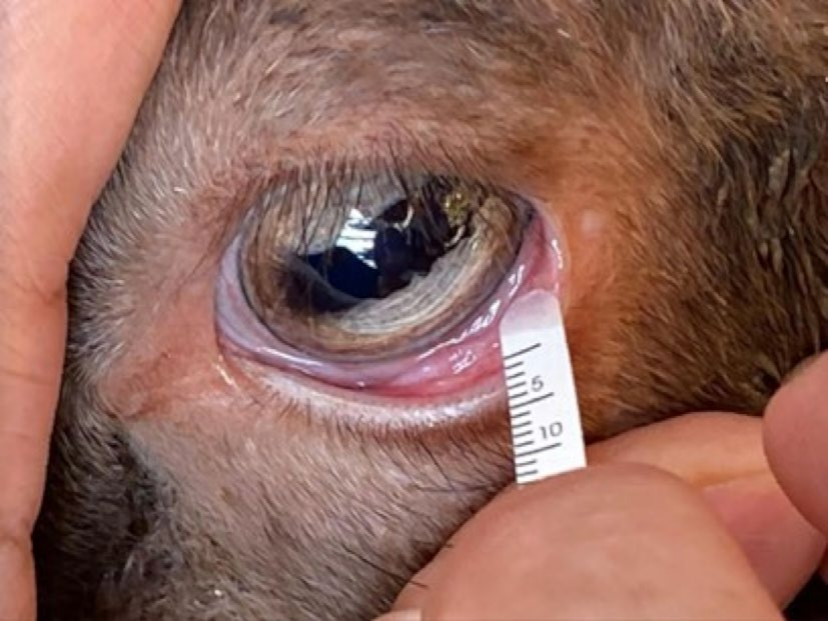
Tear fluid collection in sheep using Schirmer tear test strips.

To test the reproducibility of the collection and measurement techniques, a daily sample was collected from the left eye of each subject for five consecutive days. Samples were collected at the same time each morning (between 9:00 and 10:00 a.m.). The examiner and assistant did not wear gloves. Schirmer strips from a single lot (lot no. MIPL/A1/93, IMS Ophthalmic Strips, IMS, London, UK) were placed in the ventral conjunctival fornix without application of topical anesthesia and removed when the 5‐mm mark was reached to standardize the amount of tear fluid collected from each animal (Figure 1). Strips were stored in a labeled Eppendorf tube at room temperature until further analysis.

Two intact, dry strips were also provided for analysis. These were used as control samples to assess the elemental composition of the strips themselves.

### 2.3 Particle Induced X‐Ray Emission (PIXE)

After tear collection, Schirmer strips were dried using a nitrogen stream and then cut with a scalpel up to the first printed ink marking on the Schirmer tear test strip. From each, a standardized 2 mm segment at the proximal end (closest to the tear meniscus) was analyzed to minimize inhomogeneity and chromatographic effects. Samples were mounted on silicon wafers using double‐sided carbon tape, as the sample holder itself contained iron (Fe) and copper (Cu) impurities, and the strips were beam‐transparent. The mounted samples were placed in a high‐vacuum chamber (~10^−7^ Torr) for PIXE analysis.

**FIGURE 2:**
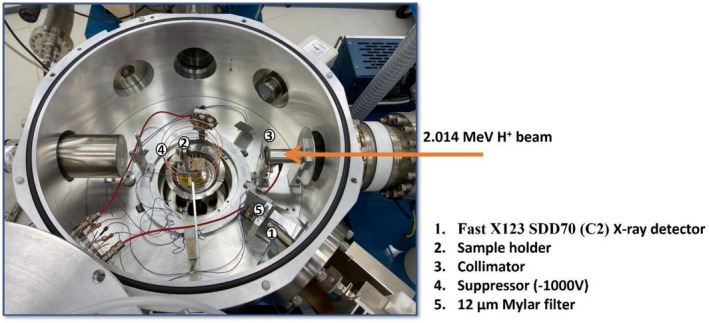
Schematic representation of the experimental setup, illustrating the spatial configuration and functional relationship between the particle accelerator, sample detector, collimator, beam suppressor, and filter.

The PIXE experiments were performed with a 1.7 MV Pelletron accelerator (National Electrostatics Corporation, Middleton, WI). Data were acquired with a Fast X123 SDD70 (C2) detector (Amptek, Bedford, MA), with a nominal surface area of 30 mm^2^, silicon (Si) crystal thickness of 500 μm, and a minimal thick silicon nitride (Si_3_N_4_) window. The energy resolution of this detector is 5.9 k‐electron volt (keV), which corresponds to the Kα X‐ray emission line transition of manganese of 135 eV (eV). The PIXE detector was positioned at 40° to the beam normal (IBM geometry) with a solid angle of 2.5 msr (msr). A filter (12 μm Mylar) was used for all measurements. Spectra were collected with a 2.014 MeV ^1^H^+^ ± 1 keV beam, with a current of ~14 nanoampere (nA) and a 1.5 mm nominal diameter of the beam. It should be noted that the portion of the sample that can be measured with conventional PIXE is limited by the size of the beam collimator (1.5 mm in diameter in this study). One electron suppressor was connected between the beam entrance and the sample holder, biased at −100 V versus ground, and a second suppressor was connected before the sample, biased at −1000 V. A normal incident beam and integrated charge (Q) of 5 μC (μC) were used for all measurements. Figure 2 depicts the experimental setup. PIXE data were analyzed using GUPIX software. Analyte concentration was determined from a calibration curve established by measuring a series of standard dry matrix samples. For the dry matrix, aqueous multi‐elemental working solutions in 1% HNO_3_ (high purity) were prepared from single element stock solutions of 1000 mg/L vanadium (V), Fe, cobalt (Co), Cu, and zinc (Zn) and by dissolution of salts (analytical grade, Sigma‐Aldrich, Germany): NaNO_3_, Mg(NO_3_)_2_ × 6H_2_O, (NH_4_)_2_HPO_4_, (NH_4_)_2_SO_4_, KNO_3_, and Ca(NO_3_)_2_ x 4H_2_O. Concentration ranges for each element were as follows: Sodium (Na) 400–5000 mg/L, magnesium (Mg) 10–100 mg/L, phosphorus (P) 50–500 mg/L, sulfur (S) 100–2500 mg/L, potassium (K) 100–2500 mg/L, calcium (Ca) 10–200 mg/L, and Fe, Co, Cu, Zn 1–50 mg/L. The concentration of V internal standard in each solution was 20 mg/L. Working solutions used for dry matrix standards (DMS) sample preparation from volumes < 50 μL were prepared with 50 mg/L V to assure good counting statistics. For DMS preparation, 50 μL of spiked samples were pipetted directly onto the pre‐cut filter paper discs (φ = 12 mm), which were mounted on a polystyrene multi‐well plate. The samples were left to dry under ambient conditions and stored in a desiccator. Blank DMS samples were prepared by the addition of 50 μL of 1% HNO_3_ solution and 50 μL of Milli‐Q water to the filter paper discs. Pure filter paper without the addition of solvent was also prepared as a blank for assessment of impurities in the substrate.

To assess the potential influence of background elemental composition in the Schirmer paper used for sample preparation, its unique spectrum was analyzed.

### 2.4 Data Analysis

Normality of the data was assessed using the Shapiro–Wilk test. As the data did not meet the assumptions of normality (*p* ≤ 0.048), the Kruskal–Wallis test followed by Dunn's post hoc test with Bonferroni correction for multiple pairwise comparisons was used to evaluate daily fluctuations in individual element concentrations over five consecutive days. A two‐sided alpha level of 0.05 was used to determine statistical significance; for multiple comparisons, a Bonferroni‐adjusted significance threshold of *p* < 0.007 was applied. Statistical analysis was performed using SigmaPlot version 14.5 (Systat Inc., Chicago, IL, USA).

## 3 Results

A total of 10 sheep was included in the study. The mean age was 62.9 ± 5.0 months (range 60–75, median 61).

Na, Ca, Cl, and Fe were present in both the Schirmer paper substrate and the tear samples. The normalized peak areas of these elements from the Schirmer strip were subtracted from each measured ovine sample spectrum. Additionally, the Schirmer strip revealed traces of Si, aluminum (Al), Cu, and chromium (Cr), which were not present in the tear samples.

**FIGURE 3:**
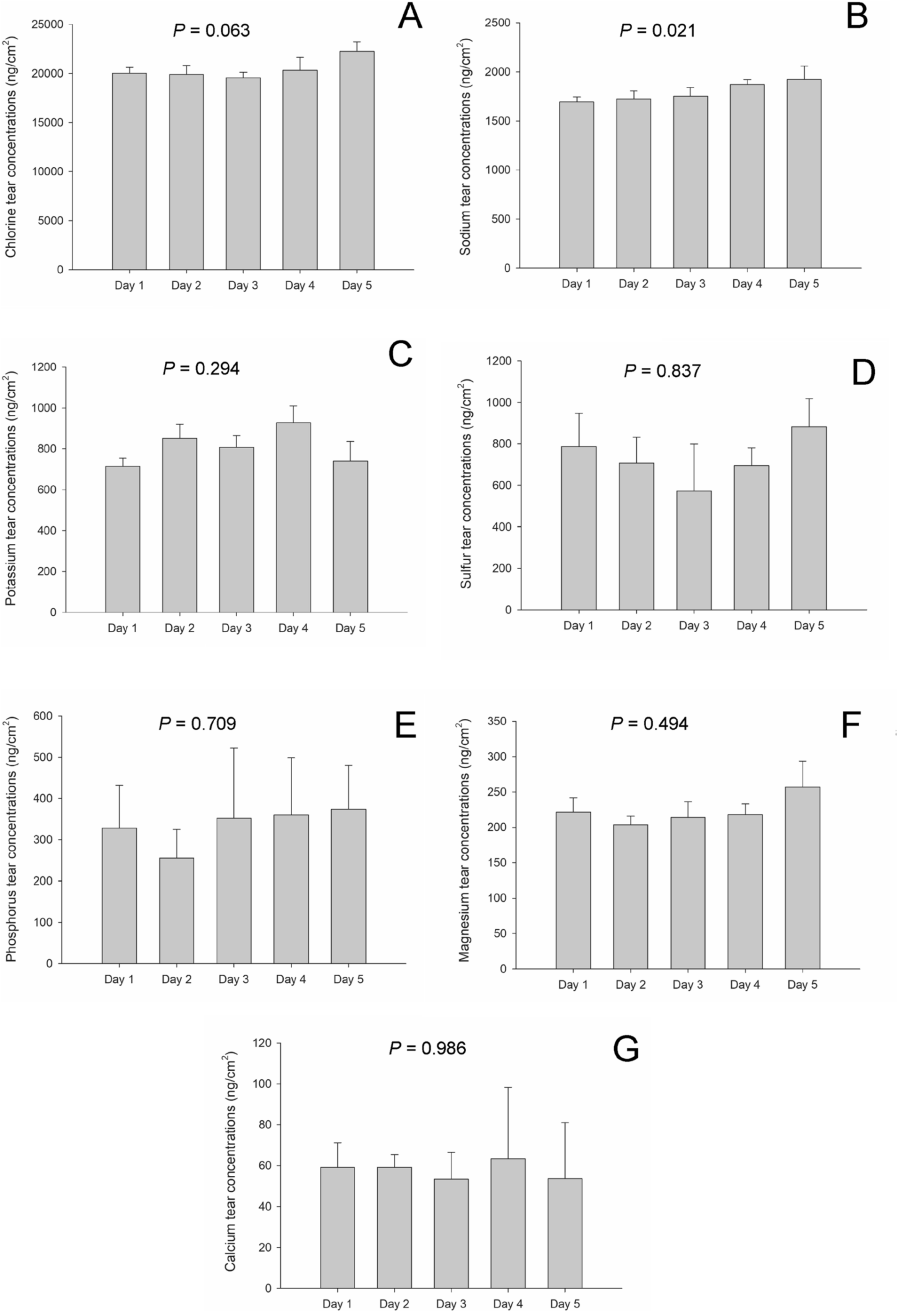
Bar charts depicting median + SEM concentrations of elements in tears of 10 ophthalmologically healthy sheep, whereby tear samples were collected and analyzed over five consecutive days. (A) Chloride; (B) sodium; (C) potassium; (D) sulfur; (E) phosphorus; (F) magnesium; (G) calcium.

**FIGURE 4:**
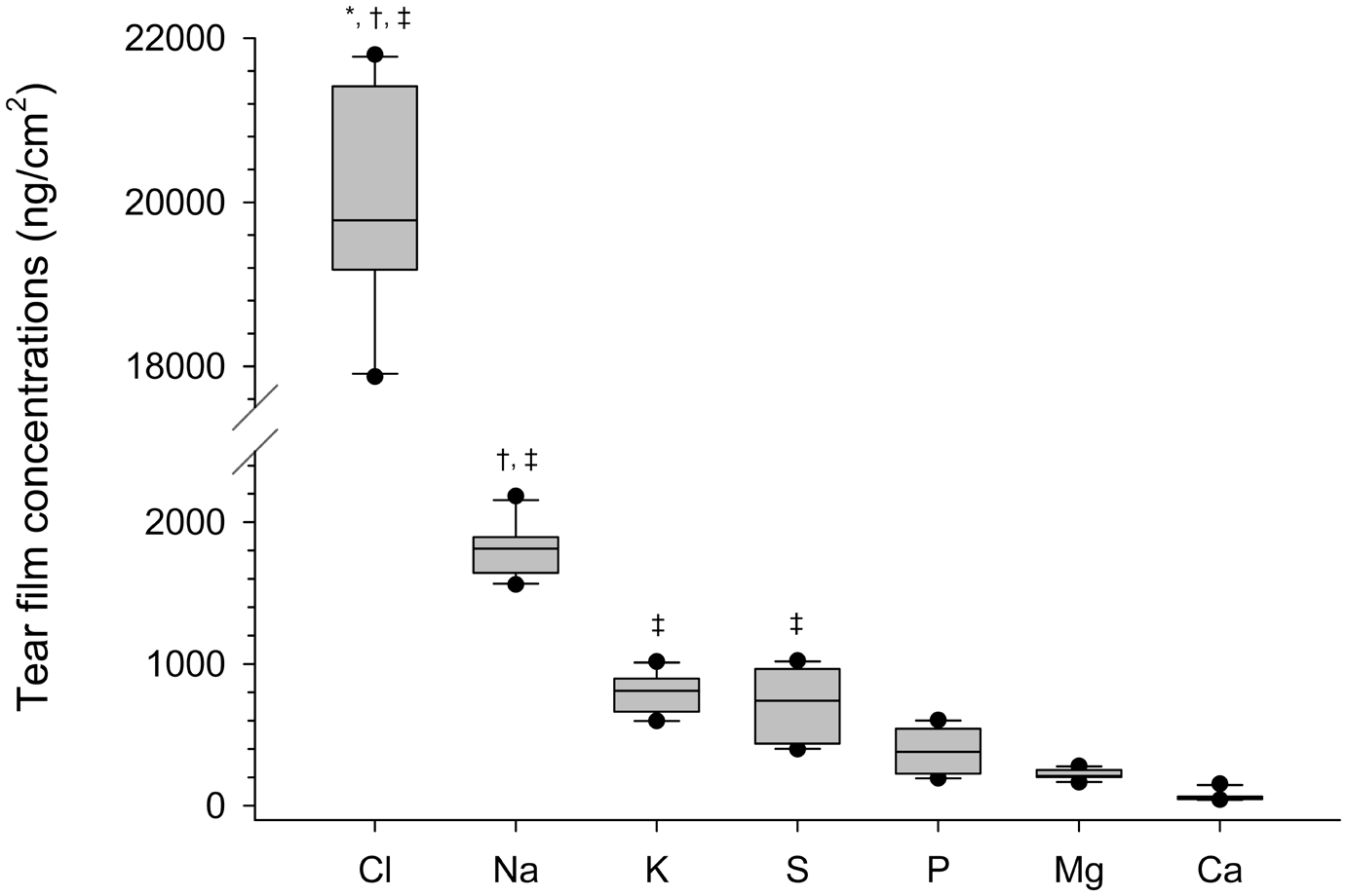
Box‐and‐whisker plots comparing the concentrations (ng/cm^2^) of elements in tears of 10 healthy sheep, including chloride (Cl), sodium (Na), potassium (K), sulfur (S), phosphorus (P), magnesium (Mg), and calcium (Ca). Each plot represents the median (horizontal line), 10th percentile (lower whisker), 25th percentile (lower limit of box), 75th percentile (upper limit of box), 90th percentile (upper whisker), and outliers (dark circles). Symbols are used to depict a significant difference (*p* < 0.007) between the concentration of a given element and the concentration of P (*), Mg (†), and Ca (‡).

Seven distinct elements were quantified in the tear samples measured over five consecutive days from the 10 sheep. Figure 3 presents the median (+SEM) daily tear film concentrations. There were no significant differences among the daily measurements for any of the elements (*p >* 0.007 after Bonferroni correction). Figure 4 presents the median (±SEM) of the five daily readings for the different elements. Listed in descending order of concentration, and as detailed in Table 1, these elements were chloride (Cl), Na, K, S, P, Mg, and Ca.

**TABLE 1:** Median, standard error of the mean (SEM), and range (minimum–maximum) of concentrations of elements (ng/cm^2^) in tears in 10 ophthalmoscopically healthy sheep, including chloride (Cl), sodium (Na), potassium (K), sulfur (S), phosphorus (P), magnesium (Mg), and calcium (Ca).

	Cl	Na	K	S	P	Mg	Ca
Median (SEM)	19 781 (421)	1813 (57)	810 (44)	740 (77)	380 (50)	210 (11)	58 (10)
Range	17 873–21 800	1559–2183	598–1017	399–1022	193–604	165–281	42–155

## 4 Discussion

In the present study, PIXE was used for the analysis of the elemental composition of tears in sheep. The results demonstrated low daily variability in elemental concentrations across the 5‐day sampling period, indicating good reproducibility under controlled collection conditions.

Schirmer strips are a widely used method for tear collection, though they are limited by the small volume of tears obtained, typically ranging from 3.4 to 10.7 μL per eye in different species [4, 5, 6, 7, 8, 9, 10, 11]. Alternative tear collection techniques include micropipettes, which can collect 5–10 μL but require precise handling and may cause reflex tearing; capillary tubes, which collect 2–20 μL but demand direct and sometimes irritating contact with the tear meniscus; and disposable syringes, which allow for larger volumes (1–60 mL) but are impractical for small species and risk contamination [12, 16, 17, 18, 19, 20]. Some studies have pooled tear samples from multiple subjects to compensate for low volumes, but this approach is not ideal for individual concentration analysis [12, 20, 21, 22].

Despite the relatively low sample volume obtained with Schirmer strips, PIXE analysis requires only a few microliters, making it an effective technique for elemental detection in low‐volume biological samples [23]. The minimum sample volume needed for PIXE is approximately 1–5 μL, depending on the concentration of elements present and the sensitivity of the detection system. This study is the first to apply PIXE for tear analysis in sheep, and we successfully detected elements with detection limits in the nanogram range, demonstrating the feasibility of this approach.

In this pilot study, we examined the tear composition of a physically normal group of sheep with a controlled habitat and diet. Sampling was performed without gloves, as the use of gloves during sampling was not reported in previous studies, which mainly examined protein, urea, glucose, and cholesterol levels and not elemental concentrations [13]. Additionally, meticulous attention was given to only soak the proximal 5 mm segment of the strip, which limited tear volume and potentially minimized variability between samples (Figure 3). Due to the capability of PIXE to detect minute concentrations of elements, a meticulous sampling technique is of paramount importance to avoid contamination and achieve accurate results.

Most previous studies of tear composition focused on lipids and proteins. Very few examined elemental composition in animal tears, and none used the PIXE method for animal tear analysis. In a study of the electrolyte composition of canine tears, Knight et al. [22] utilized the VITROS 5,1 FS Chemistry Analyzer, which required large volumes of 500 μL of tears from each eye for analysis. Of the few electrolytes examined, including Na, Cl, K, Ca, P, and Mg, Na and Cl had the highest concentrations, similar to the results of the present study. Similar to our study, in canine tears K concentrations were significantly higher than Ca, P, and Mg concentrations.

Oriá et al. [24] evaluated the electrolyte composition of bird and reptile tears, particularly Cl, P, Fe, Na, K, and Ca. Unlike our study, tear samples were pooled from each species to generate a sufficient volume for analysis with commercial colorimetric kits. The authors found that the electrolyte balance was similar in all species evaluated, with higher Na, Cl, and Fe concentrations. In species studied by Oria et al., Fe concentrations were higher than concentrations of Na or Cl.

Several other studies examined the elemental composition of human tears using various methods. In a previously published study by our group, human tear elemental analysis with PIXE identified differences in Na, Cl, K, Al, Fe, Cr, Cu, and titanium (Ti) between individuals living in rural and urban environments [25]. These findings were consistent with an analysis of concentrations of Cr, Cu, selenium (Se), arsenic (As), Zn, rubidium (Rb), barium (Ba), lead (Pb), and Co conducted by Semeraro et al. [26] using Inductively Coupled Plasma Mass Spectrometry (ICP‐MS). The authors suggested that the differences in tear elemental concentrations between rural and urban populations may be due to differences in environmental and nutritional factors [26]. Kaplan et al. [27] applied energy dispersive spectroscopy (EDS) to analyze the elemental composition of human tears in a clinic and home setting, and detected a total of 19 metallic [Al, Cu, Zn, Ti, Cr, Mg, Fe, gold (Au), molybdenum (Mo), nickel (Ni), silver (Ag)] and nonmetallic (Na, Cl, K, S, Si) elements. Many of the elements detected in human tears, including Au, Mg, Cu, Zn, Ti, Ni, Ag, Cr, As, Se, Rb, Ba, Pb, and Co, were not detected in our study in sheep tears, and one (P) was detected in sheep tears but not in human tears. The differences among the studies might be attributable to the different detection techniques used in addition to innate interspecies differences, environmental exposures, or nutrition.

PIXE, ICP‐MS, and EDS have inherent differences in terms of sample volume requirements, detection limits, and analytical capabilities. PIXE requires a minimal sample volume of 1–5 μL and has detection limits in the nanogram per square centimeter (ng/cm^2^) range, making it particularly useful for analyzing small biological samples like tears in a non‐destructive manner [23]. In contrast, ICP‐MS is highly sensitive, with detection limits reaching the parts per trillion (ppt) to parts per billion (ppb) range [26], but it requires significantly larger sample volumes (≥ 100 μL) and involves extensive sample preparation, including digestion, which may affect tear film analysis [4]. EDS, while capable of detecting a broader range of elements, has detection limits typically in the parts per million (ppm) range, making it less sensitive than PIXE and ICP‐MS for trace element analysis [27]. Additionally, EDS is more suited for solid‐state analysis and has lower sensitivity for lighter elements [25]. The broader elemental detection reported in human tears using EDS may be attributed to differences in sample matrix, preparation, and instrumental capabilities rather than an indication that PIXE is less sensitive or accurate.

The limitations of the present study include the relatively small sample size, which may affect the generalizability of the findings and should be addressed in future studies with larger cohorts. Additionally, all animals included were of a single breed (Assaf‐Afec crossbred sheep), raised under uniform conditions. As such, the results may not be directly applicable to other breeds or species without further validation.

Future studies should aim to compare different tear collection methods to determine whether sampling techniques influence the results and to assess potential differences in elemental composition when using different methods on the same group of animals. Investigating how various sampling methods affect elemental binding to the substrate would also provide valuable insights. Furthermore, evaluating dry strips handled with and without gloves would serve as an important validation step to assess potential contamination from gloves, which should be considered in future research to enhance the reliability of the sampling process. Finally, further studies are needed to determine the potential effects of species, age, gender, habitat, diet, diurnal changes, and systemic or ophthalmic diseases on the elemental composition of sheep tears.

## 5 Conclusion

This study successfully demonstrated the use of PIXE for detecting and analyzing the elemental composition of sheep tears, providing insights into potential applications in veterinary research. No significant differences in element concentrations between individual sheep were noted over time. The findings underscore the utility of PIXE analysis with implications for the extension of the methodology to other species.

## Ethics Statement

This study complies with the ARVO Statement for the Use of Animals in Ophthalmic and Vision Research and was approved by the Institutional Animal Care & Use Committee of the Volcani Center (Rishon LeZion, Israel; permit no. 828/19).

## Conflicts of Interest

The authors declare no conflicts of interest.

## Data Availability

The data that supports the findings of this study will be made available upon reasonable request from the corresponding author.
